# 肺干细胞和肺癌干细胞的研究进展

**DOI:** 10.3779/j.issn.1009-3419.2015.10.06

**Published:** 2015-10-20

**Authors:** 荟菁 尹, 炯 邓

**Affiliations:** 1 200025 上海, 上海交通大学医学院病理生理系, 细胞分化与凋亡教育部重点实验室 Department of Pathophysiology, Key Laboratory of Cell Differentiation and Apoptosis of Chinese Ministry of Education, Shanghai Jiao Tong University School of Medicine, Shanghai 200025, China; 2 200025 上海, 上海交通大学肿瘤微环境与炎症上海市重点实验室 Shanghai Key Laboratory for Tumor Microenvironment and Inflammation, Shanghai Jiao Tong University School of Medicine, Shanghai 200025, China; 3 200030 上海, 上海胸科医院转化医学中心 Translation Medicine Center, Shanghai Chest Hospital, Shanghai Jiao Tong University, Shanghai 200030, China

**Keywords:** 肺干细胞, 肺肿瘤, 表面标志, 信号通路, 靶向治疗, Lung stem cells, Lung neoplasms, Surface markers, Signal pathway, Targeted therapy

## Abstract

癌干细胞是目前癌症研究的热点之一。肺癌干细胞与正常肺干细胞有许多共同之处, 包括自我更新能力和多分化潜能。许多癌干细胞分子标志为肺癌干细胞所共有, 如CD133、CD44、乙醛脱氢酶(aldehyde dehydrogenase, ALDH)以及ATP结合转运蛋白G超家族成员2(ATP-binding cassette sub-family G member 2, ABCG2)。肺癌干细胞的扩增与作用不仅受胚胎干细胞途径如Notch、Hedgehog和Wnt调控, 也受肿瘤信号途径如表皮生长因子受体(epidermal growth factor receptor, EGFR)、信号传导转录激活因子3(signal transducer and activator of transcription 3, STAT3)和磷脂酰肌醇3激酶(phosphatidylinositol 3 kinase, PI3K)等的调控。由于癌干细胞在肿瘤复发、转移和耐药性等方面发挥着重要作用, 揭示肺癌干细胞与正常干细胞的区别, 鉴定并靶向癌干细胞特异性表面标志物及其介导的信号通路, 将有望改善肺癌治疗效果和提高患者生存率。

## Abstract (en)

Cancer stem cells (CSCs) are emerging as a hot topic for cancer research.Lung CSCs share many characteristics with normal lung stem cells (SCs), including self-renewal and multi-potency for differentiation.Many molecular markers expressed in various types of CSCs were also found in lung CSCs, such as CD133, CD44, aldehyde dehydrogenase (ALDH) and ATP-binding cassette sub-family G member 2 (ABCG2).Similarly, proliferation and expansion of lung CSCs are regulated not only by signal transduction pathways functioning in normal lung SCs, such as Notch, Hedgehog and Wnt pathways, but also by those acting in tumor cells, such as epidermal growth factor receptor (EGFR), signal transducer and activator of transcription 3 (STAT3) and phosphatidylinositol 3 kinase (PI3K) pathways.As CSC plays an critical role in tumor recurrence, metastasis and drug-resistance, understanding the difference between lung CSCs and normal lung SCs, identifying and targeting CSC markers or related signaling pathways may increase the efficacy of therapy on lung cancer and improved survival of lung cancer patients.

肺癌的致死率为癌症中之最。肺癌就病理表型可分为两大类:小细胞肺癌和非小细胞肺癌。非小细胞肺癌约占肺癌总数的85%, 主要有肺鳞癌和肺腺癌。肺癌患者的生存率低, 手术或放化疗治疗之后容易复发, 目前认为最主要的原因是癌干细胞的存在^[1]^。癌干细胞理论认为, 癌干细胞虽然在肿瘤细胞中仅占小部分, 但该细胞亚群具有癌症发生能力, 具有自我更新、多分化潜能, 是癌症发生发展的主要原因。该理论已被大量实验结果支持^[2]^, 如肿瘤自我更新、肿瘤异质性、肿瘤治疗后复发以及对传统化疗药物的抗性等, 这些现象均可用癌干细胞理论加以诠释。

癌干细胞首次发现于人血液型癌症, 继而发现于实体瘤中。有研究人员从小细胞肺癌和肺腺癌患者的组织样本中分离出一群具有克隆形成能力的细胞(其占细胞总数的 < 1.5%), 将这些细胞注入裸鼠体内, 可形成与原肿瘤相似的表型^[3]^。目前有许多方法可用于癌干细胞的研究, 如利用癌干细胞表面特异性抗原(如CD133、CD44、ALDH、ABCG2), 通过流式细胞技术, 可以分选出癌干细胞。干细胞具有将Hoechst 33342荧光染料泵出胞外的能力, 利用该特性, 通过流式分析技术, 可分选出侧群细胞(side population, SP)或富集干细胞群。侧群细胞具备很强的自我更新能力, 其端粒酶活性较正常细胞高^[4]^, 而保持高活性的端粒酶是癌细胞永生化的一个标志。癌干细胞具有抗化疗药物的能力, 或耐药性, 经药物处理依然能够存活下来细胞, 具备许多类似干细胞特性, 包括更强的克隆形成能力, 表达CD133, 并富集在侧群细胞中^[5]^。

## 1 肺干细胞

为探索肺癌干细胞, 人们首先从正常肺干细胞着手。肺组织结构复杂, 分为呼吸部和换气部, 呼吸部主要包括气管、支气管、细支气管; 换气部主要是肺泡。不同解剖结构有相应的肺干细胞, 以维持该结构的功能。由于肺上皮更新较慢, 证实肺干细胞较其它组织更为困难。因此, 利用肺损伤模型研究肺干细胞的特性是较为常用的方法。肺干细胞的研究进展有如下几个方面(从肺近端往远端顺序进行):①在肺近端(包括气管和支气管)气道基底细胞中驻有一亚群细胞(表达Keratin-5^[6]^和Keratin-14^[7]^), 其在损伤后能迅速增殖, 并呈现恶性表型。有报道, 鳞癌频繁地出现在气管的近端, 并与一类表达Keratin-5的细胞紧密相关^[8]^。②在距离气管的稍远处(气管中部)驻留着一类Clara细胞。Clara细胞具有始祖细胞的功能, 在氧介导的肺损伤过程中, 可分化成分泌细胞和纤毛细胞, 以保护支气管免受进一步损伤^[9]^。不过, 虽然Clara细胞具有多分化的潜能, 但大多数Clara细胞不具备自我更新的能力^[10]^。③定位于肺细支气管(中部和较远端)的神经内分泌细胞, 他们能够产生特殊的神经多肽并形成簇状结构, 被称为神经小体。细支气管受损后, 修复过程会引起神经内分泌细胞增殖异常^[11]^。然而, 神经内分泌细胞不具备多分化的潜能^[12]^。小细胞肺癌频发于气管支气管的中部, 癌细胞表达干细胞标志物CD44^[8]^, 还表达Ca基因相关的肽, 具有神经内分泌细胞的特征^[12]^。④在气管远端, 尤其在细支气管末端和腺泡连接处, 存在一群支气管肺泡干细胞(bronchioalveolar stem cell, BASC), 其在肺组织损伤后会产生多种细胞亚群^[13]^。有研究^[13, 14]^表明, 肺腺癌和细支气管腺泡癌与BASC的异常增殖紧密相关。但目前还没有直接证据表明, 肺肿瘤细胞起源于BASC细胞。此外, 人肺组织具有一个干细胞池, 表达c-kit抗原, 这类干细胞在体内体外已被证实具有自我更新、克隆形成以及多分化潜能^[15]^。总之, 在肺组织的不同部位驻有不同种类的肺干细胞群, 以分化替代该部位受损细胞。

## 2 肺干细胞与肺癌干细胞的关系

目前一个备受关注的问题是, 癌干细胞起源于何种细胞？有假说认为, 在快速分裂的组织中, 正常细胞会因遗传突变而发生恶性转化(transformation)。累积有各种遗传突变的恶性转化型干细胞是癌干细胞的起源^[16]^。然而, 肺癌干细胞是否直接来源于肺干细胞？目前并无确凿的证据。肺干细胞本身的作用是维持肺的正常功能。但是, 肺损伤等刺激可导致肺干细胞的异常扩增, 此过程中可能会累积遗传突变, 使之具有部分癌干细胞的特性。但是肺干细胞如何获得肺癌干性以及如何演变为癌干细胞仍然不清。例如, BASC细胞表达SPC和CC10, 被认为很可能是肺腺癌的起源细胞。但根据种系标记追踪方法发现, 肺腺癌仅表达SPC, 不表达CC10。这说明, BASC细胞还不是肺腺癌的直接起始细胞。

## 3 干细胞微环境与肿瘤微环境

干细胞亚群的存在需要“壁龛”(niche)来维持, Niche是能调控干细胞自我更新和分化的微环境。Niche可有多种细胞, 如间充质细胞, 包括内皮细胞、成纤维细胞以及细胞外基质(extracellular matrix, ECM)。那么, 干细胞的调控是受自身还是受组织微环境调控呢？这个问题目前还不十分清楚, 但很可能与两者都有关。一般情况下, 肺干细胞处于休眠状态。只有当组织受损时, 肺干细胞和始祖细胞才会因之而扩增, 以修复受损组织。实体瘤的微环境包括不同类型的间充质细胞, 如血液和淋巴循环中的内皮细胞、炎症细胞和成纤维细胞等。这些间充质细胞被癌细胞所招募, 能促进肿瘤生长、迁移和浸润^[17]^。例如, 成纤维细胞可产生不同的趋化因子, 调控肿瘤的生长、迁移以及促血管生成^[18, 19]^。有研究^[20]^发现, 将肺癌细胞Calu-3与从非小细胞肺癌患者的组织样品中分离出间充质细胞(hLc-MCs)共培养, 其对裸鼠的致瘤性要比单独培养的Calu-3细胞更强。原位染色发现, hLc-MCs驻留在由Calu-3形成的腺癌周围。这提示, 间充质细胞对癌细胞成瘤有促进作用。另一方面, 在肺上皮细胞特异表达*K-Ras*基因的转基因小鼠的肺组织中, BASC细胞被发现异常性扩增, 且与肺肿瘤发生密切相关。这提示, 肺上皮细胞中高表达K-Ras足以导致肺干细胞群的异常扩增^[14]^。研究发现, 细胞干性还可由上皮细胞间质转型(epithelial-mesenchymal transition, EMT)诱发。EMT指上皮细胞(包括细胞间粘附及极性等)向间质细胞(包括细胞运动和抗凋亡)的转换过程。有报道, 用烟草致癌剂处理永生化人支气管上皮细胞4周, 会诱导出EMT特征(包括E-cadherin的下调和ZEB1的上调)及细胞形态的改变; 用该致癌剂处理12周之后, 这些细胞会表现出某些干细胞的特征, 包括表达CD44^+^/CD24^-^, 及具备细胞成球能力^[21]^。一氧化氮(nitric oxide, NO)在许多肿瘤中均有较高的表达水平。最新研究^[22]^发现, NO可诱导非小细胞肺癌细胞H292和H460发生EMT样变化, 如表达干细胞相关蛋白CD133和ALDH1A1, NO还可诱导Cav-1表达上调, 而Cav-1的上调可导致这些癌细胞抗凋亡、迁移和浸润性增强。总之, 肿瘤微环境具有促发癌干细胞形成的能力。而肿瘤干细胞中的遗传突变也会导致癌干细胞的扩增。

## 4 肺癌干细胞的表面标志

### 4.1 CD133

CD133是细胞表面糖蛋白, 包括5次跨膜结构域和2个大的糖基化外环。有研究^[23]^表明, 在非小细胞肺癌患者的组织样本以及肺癌细胞系中, CD133阳性亚群要比CD133阴性亚群细胞具有更强的自我更新、肿瘤发生和耐药性, 并表达更高水平的Oct-4(胚胎干细胞相关转录因子)。此外, 除化疗药物顺铂^[24]^, 低氧诱导因子1α(hypoxia inducible factor 1α, HIF1α)/HIF2α也可诱导肺癌细胞中Oct4和Sox2的表达增加, 后二者可直接结合到CD133启动子上, 增加CD133的表达^[25]^。尽管CD133在不同类型癌症中的表达意义仍有争议, 但在肺癌发生发展过程中, CD133被认为具有重要作用。有研究^[26]^在数百例非小细胞肺癌组织样本中发现, CD133主要集中在肿瘤细胞的胞浆和胞核区, 其上调程度与肿瘤大小、分化程度以及肿瘤等级呈显著相关。统计结果显示, 胞浆和胞核中CD133的上调均可独立作为预后不良的指标。

### 4.2 CD44

CD44是透明质酸受体, 在几乎所有类型的肿瘤中均有表达。从患者恶性胸腔积液获得的原代细胞, 经分选可获得CD44阳性和阴性细胞。对比这两种细胞可发现, CD44阳性细胞中BMI1和hTERT的转录水平显著上调, 体外克隆形成能力和体内致瘤能力也均显著增高。另外, 在恶性胸腔积液来源的细胞以及非小细胞肺癌细胞H2122中, 发现有染色体畸变和1p36的缺失。这导致位于1p36的抑癌基因miR-34a的表达显著下降。而过表达miR-34a能够减弱CD44阳性细胞的干性表型。在临床肺癌患者样品中, CD44与另一个干细胞相关基因*ALDH*有共定位现象, 这种共定位几率在肺鳞癌中较高^[27]^。

### 4.3 ALDH

ALDH是乙醛脱氢酶, 它在正常干细胞的乙酰辅酶氧化反应和分化调控上发挥重要作用。在大多数的非小细胞肺癌细胞中, ALDH的活性被发现显著增强, 这与ALDH1A1表达上调显著相关。与ALDH阴性细胞相比, ALDH阳性细胞在细胞自我更新、克隆形成及致瘤能力方面均显著增强。Notch通路的激活与ALDH阳性细胞有关, 抑制Notch通路可导致ALDH阳性细胞数目减少, 克隆形成能力下降。临床样品分析表明, ALDH1A1的表达与肺癌患者的预后不良相关^[28]^。2012年, ALDH-1被确立为人肺腺癌特异性标志物^[29]^。此外, 肺癌患者对Gefitinib(EGFR酪氨酸激酶特异性抑制物)的耐药性与ALDH1A1的表达上调有关^[30]^。

### 4.4 ABCG2

ABCG2广泛分布于正常组织中, 在干细胞中呈高表达。ABCG2富集在侧群细胞中, 能将Hoechst 33342染料泵出胞外。这一活性使ABCG2的功能与癌细胞的抗药性联系在一起。ABCG2在肺癌细胞中的高表达可能与转录因子Sp1和Sp3有关, 因为二者可以结合到ABCG2的启动子区域, 促进ABCG2的转录。用ABCG2抑制物Mithramycin A(能阻止Sp1与ABCG2的转录启动区结合)处理A549细胞, 可使侧群细胞减少, 成球能力下降, 并增加其对化疗药物顺铂的敏感性^[31]^。用烟草类浓缩物处理肺癌细胞可导致ABCG2及转录因子AhR、Sp1和Nrf2的表达显著上调。可见, 吸烟诱发肺癌的部分原因可能是通过上调ABCG2表达来实现的^[32]^。

### 4.5 CD133和ALDH

一项基于对205例非小细胞肺癌患者的研究^[33]^发现, 肺肿瘤切除后早期如出现ALDH1A1和CD133共表达的现象, 患者的无病生存率和总存活率最低。这个结果将ALDH1A1和CD133功能联系在一起, 其机理还有待进一步的研究去揭示。

### 4.6 CD44和ALDH

从临床肺癌患者术后组织或恶性胸腔积液中分离细胞, 进行原代培养(第1-5代), 经分选可获得CD44∕ALDH(双高)亚群细胞。相对于其他组细胞, 这群细胞的干性相关基因以及EMT相关转录因子的表达均显著上调。这些细胞表现出高迁移能力, 细胞周期阻滞在G_2_期/M期, 并且有显著增强的致瘤性。用Hedgehog通路转录因子GLI1的抑制剂Cyclopamine, 或Notch通路转录因子HES1的抑制剂RO4929097, 均能显著减少CD44∕ALDH(双高)亚群细胞的数目。用EGFR抑制剂Gefitinib处理细胞会导致CD44∕ALDH(双高)细胞数目增加。这说明, CD44∕ALDH(双高)细胞具有抗药性。使用ALDH抑制剂DEAB, 同时敲低CD44的表达, 可增加细胞对Gefitinib的敏感性, 并下调其他干性相关基因的表达。在临床肺癌患者中, CD44/ALDH高表达与无病生存率的降低紧密相关^[34]^。这些结果表明, CD44和ALDH共表达在维持细胞干性(维干)、肿瘤发生以及耐药性方面有重要作用, 其机制可能是同时启动了Hedgehog和Notch通路。

### 4.7 CD166

最近有报道, 在非小细胞肺腺癌患者样本中, 通过CD166可富集肺癌干细胞。CD166阳性细胞群可使裸鼠致瘤。该亚群细胞的甘氨酸/丝氨酸代谢途径被改变, 这项发现与患者样本中甘氨酸脱羧酶以及甘氨酸/丝氨酸代谢酶的高表达相一致^[35]^。不过, 作为肺癌干细胞标记, CD166目前还没有得到广泛认同。

### 4.8 Sox2和Oct4

Sox2和Oct4是调控胚胎干细胞自我更新和分化的转录因子。有研究^[36]^发现, 在非小细胞肺癌患者的组织样本中, Sox2和Oct4仅表达在癌细胞核内, 而在癌旁中不表达。Oct4的高表达与非小细胞肺癌的低分化和预后不良紧密相关。因此, Sox2和Oct4有望作为非小细胞肺癌患者诊断分子标记或治疗的靶标。

## 5 肺癌干细胞的调控机理

### 5.1 Notch通路

在研究化疗耐药机制时研究人员发现, 用低剂量顺铂处理肺癌细胞H460和H661细胞, 会显著上调CD133、ABCG2和ABCB1的表达。同时, 这些细胞会表现出对地塞米松和紫杉醇的耐药性。顺铂诱导CD133的表达是通过Notch1的活化, 用γ-分泌酶抑制剂DAPT预处理细胞, 可增加细胞对地塞米松和紫杉醇的敏感性。动物实验发现, 顺铂可使小鼠体内剪切型Notch1(激活型)和CD133表达的显著增加。临床样本分析发现, 顺铂治疗后又复发肺癌患者中, 活化型Notch1/CD133阳性率占50%^[37]^。这些结果表明, Notch通路在调控肺癌干细胞上有重要作用。

### 5.2 Hedgehog通路

有研究发现, Sox2可被PKC1磷酸化, 两者被招募到Hedgehog酰基转移酶(Hedgehog acyltransferase, HHAT)的启动区上, 促进HHAT的表达。HHAT的表达对于维干有重要作用。有研究^[38]^发现, PKC1-Sox2-HHAT信号轴的激活对于原代肺鳞癌细胞的维干功能发挥着重要作用。

### 5.3 Wnt通路

EMT导致Wnt通路的激活, Wnt通路活化后对于癌干细胞的重编和维干发挥重要作用。研究^[39]^表明, EMT可诱导复合物β-catenin/E-cadherin/Sox15向复合物β-catenin/Twist1/TCF4转化。Twist1与β-catenin的结合使β-catenin/TCF4复合物获得转录活性, 进而诱导癌干细胞标记ABCG2的转录。该发现的临床意义在于, 可将核内高β-catenin/核内高Twist1/低E-cadherin/低Sox15/高CD133的细胞亚群作为人肺癌的诊断标志物。

### 5.4 表皮生长因子受体(epidermal growth factor receptor, EGFR)-Notch通路

用EGFR酪氨酸激酶抑制剂Erlotinib治疗有*EGFR*突变的肺癌患者时, 常常会出现耐药, 其原因可能是癌干细胞的存在和扩增。那么癌干细胞是通过什么通路发挥作用呢？研究^[40]^发现, 用Erlotinib处理含有*EGFR*突变的肺癌细胞株会导致ALDH亚群的细胞增加, 且克隆形成效率增加, 即干性增加, 其原因是Erlotinib激活了Notch3通路。这提示, 使用Erlotinib治疗时, 若联合使用靶向Notch3通路的抑制剂, 或许可降低癌细胞对Erlotinib的耐药。

### 5.5 STAT3通路

除了EGFR通路, STAT3通路在干性调控上也发挥了一定的作用。从非小细胞肺癌细胞中分选出ALDH阳性细胞, 其ALDH1A3表达显著上调。敲低ALDH1A3可显著下调这些细胞中ALDH的活性、克隆形成能力和致瘤性。这说明, ALDH1A3亚型对ALDH功能的发挥十分重要。ALDH阳性细胞群中STAT3的活性显著上调, 抑制STAT3活性或其激活子EZH2均可抑制ALDH阳性细胞的数目和克隆形成能力。这说明, STAT3信号的激活对于ALDH的活性及增强细胞干性有重要作用。由于ALDH1A3在不吸烟的女性且是分化程度高的肺腺癌中的表达显著上调, 提示靶向STAT3或许是治疗这类癌症患者的良策^[41]^。

### 5.6 JNK通路

EphA2是酪氨酸激酶受体的成员, 他在非小细胞肺癌中的表达显著上调, 并与预后不良紧密相关。有研究^[42]^发现, 抑制EphA2的表达会导致ALDH阳性细胞的数目减少, 悬浮培养形成微球的能力下降, 以及对裸鼠的致瘤能力也下降。EphA2促进细胞增殖需要JNK信号通路的活化。这提示, JNK通路参与肺癌干细胞的调控。

### 5.7 PI3K通路

*FBLN3*是目前发现的抑癌基因。有研究^[43]^发现, 在恶性高转移性的肺癌中, FBLN3的启动区呈高度甲基化。过表达FBLN3可抑制EMT和细胞成球, 下调Sox2和β-catenin, FBLN3抑制干性是经IGF1R/PI3K/AKT/GSK3β介导。因此, PI3K/AKT通路也参与了肺癌干细胞的调控。

## 6 靶向肺癌干细胞的治疗策略

小细胞肺癌恶性程度高, 治疗后很快复发, 其最主要的原因是癌细胞对药物产生抗药性。而抗药性多数由癌干细胞引起。体内体外的实验都证实, 小细胞肺癌细胞中CD133的高表达与其抗药性和致瘤性紧密相关。临床上, 小细胞肺癌患者接受化疗后, 其CD133的表达也显著上调。CD133阳性小细胞肺癌细胞会表达一种促有丝分裂的神经肽受体。用该受体的拮抗剂SP-G可抑制CD133阳性小细胞肺癌细胞的生长, 并诱导凋亡。鉴于SP-G已经是临床上治疗小细胞肺癌的Ⅰ期药物, SP-G可能是对CD133阳性、有化疗抗药性的小细胞肺癌患者进行个体化治疗的有效药物^[44]^。

对于非小细胞肺癌晚期的治疗, 目前主要采用以顺铂为主的联合用药。如这些患者的肿瘤中发现有*EGFR*激活型突变, 用顺铂加EGFR酪氨酸激酶抑制剂进行治疗, 其对肿瘤的抑制效果可达80%。遗憾的是, 患者不久还会出现肿瘤的复发及对所用药物的抗药性, 其原因可能是药物只能杀死非干细胞, 而对干细胞无效。最近, 有研究人员从非小细胞肺癌患者组织中分离出细胞, 进行原代培养(或使用非小细胞肺癌细胞株), 发现在未分化的培养条件下所有细胞均呈现共同特征:①CD133/EpCAMS双阳性细胞显著增加; ②EGFR的活性显著增强。有意思的是, CD133/EpCAMS双阳性细胞对顺铂的耐药性显著增强, 但对EGFR激酶抑制剂Afatinib的敏感性增加。这提示, 对CD133/EpCAMS双阳性干细胞, 使用Afatinib与顺铂联合用药可缓解这些细胞的抗药性^[45]^。Erlotinib处理肺癌细胞会导致ALDH活性的增加, 而ALDH与细胞干性增强紧密相关。Silibinin是一种具有生物活性的黄酮木脂素, 具有抗氧化功能, 他可逆转这些细胞对Erlotinib的抗药性。因此, Silibinin有望成为与Erlotinib联合使用的候选药物^[46]^。AST1306是EGFR和ErbB2抑制物, 目前已经用于肺癌临床Ⅰ期治疗。研究^[47]^发现, AST1306可抑制ABCG2对药物的泵出能力, 增加药物在胞内的累积, 借此提高药效。Galectin-3是肺癌干细胞中一种新发现的候选维干分子。在人肺癌H1299细胞的成球中, Galectin-3表达上调。随着细胞球的传代, 其表达持续增加。在人肺癌细胞A549中过表达Galectin-3可使本身成球效率低的细胞获得较高的成球能力。H1299形成的球体中β-catenin的活性显著上调。抑制Galectin-3的表达可显著抑制β-catenin的活性及干性相关的基因(包括Oct4、Sox2、Nanog和CD133)的表达, 同时还能抑制干性相关表型, 如成球能力、致瘤性和抗药性^[48]^。总之, 靶向癌干细胞的策略被认为是克服肿瘤抗药性的关键。

## 7 问题和展望

尽管对肺干细胞和肺癌干细胞的研究已经取得了很多进展, 但仍有许多问题有待解决:①缺乏能鉴定肺癌的特异性标记或不同亚型的肺癌干细胞标记。已知的肿瘤干细胞的分子标记(如CD133、CD44、ALDH以及ABCG2)在鉴定肺癌干细胞方面仅有参考意义。对于诊断、治疗还需要发现具有肺癌干细胞的特异性表面标志物, 这样既有利于分离、研究, 也有利于靶向治疗。②缺少合适的动物研究模型。肺癌干细胞的深入研究很大程度上取决于合适的肺癌动物模型。研究不同亚型肺癌中肺癌干细胞的作用以及研究其他的肺部疾病(包括肺气肿、慢阻肺等疾病)中肺干细胞的作用, 都需要建立相应合适的动物模型。因此, 建立新型、与人疾病相似的疾病动物模型, 对阐明肺干细胞向肺癌干细胞恶性转化机制的研究, 至关重要。③缺少清除残留肺癌干细胞的有效方法。虽然靶向治疗加上传统治疗方法(包括手术、放化疗等)能够清除大部分肿瘤或抑制肿瘤生长, 但是残存的肺癌干细胞仍能使肿瘤复发, 而肺癌干细胞对放化疗更为耐受。针对已知的肺癌干细胞表面标志物或肺癌干细胞通路, 小分子靶向治疗的效果还十分有限, 且可选择的治疗方法有限。

总之, 探明肺干细胞的调控机理, 鉴定肺癌细胞的起源, 不仅能揭示肺癌的发生机理, 且将有助于发现肺癌干细胞的特异性标记, 提供治疗肺癌抗药性的新靶点, 其很可能成为提高肺癌治疗的关键。
